# Classification-augmented survival estimation (CASE): A novel method for individualized long-term survival prediction with application to liver transplantation

**DOI:** 10.1371/journal.pone.0315928

**Published:** 2025-01-17

**Authors:** Hamed Shourabizadeh, Dionne M. Aleman, Louis-Martin Rousseau, Katina Zheng, Mamatha Bhat

**Affiliations:** 1 Department of Mechanical & Industrial Engineering, University of Toronto, Toronto, ON, Canada; 2 Department Mathematics & Industrial Engineering, Polytechnique Montréal, Montréal, QC, Canada; 3 Division of Gastroenterology & Hepatology, University of Toronto, Toronto, ON, Canada; State University of New York at Oswego, UNITED STATES OF AMERICA

## Abstract

Survival analysis is critical in many fields, particularly in healthcare where it can guide medical decisions. Conventional survival analysis methods like Kaplan-Meier and Cox proportional hazards models to generate survival curves indicating probability of survival v. time have limitations, especially for long-term prediction, due to assumptions that all instances follow a general population-level survival curve. Machine learning classification models, even those designed for survival predictions like random survival forest (RSF), also struggle to provide accurate long-term predictions due to class imbalance. We improve upon traditional survival machine learning approaches through a novel framework called classification-augmented survival estimation (CASE), which treats survival as a classification task that ultimately yields survival curves, beginning with dataset augmentation to improve class imbalance for use with any classification model. Unlike other approaches, CASE additionally provides an exact survival time prediction. We demonstrate CASE on a liver transplant case study to predict >20 years survival post-transplant, finding that CASE dataset augmentation improved AUCs from 0.69 to 0.88 and F1 scores from 0.32 to 0.73. Compared to Kaplan-Meier, Cox, and RSF survival models, the CASE framework demonstrated better performance across various existing survival metrics, as well as our novel metric, mean of individual areas under the survival curve (mAUSC). Further, we develop novel temporal feature importance methods to understand how different features may vary in survival importance over time, potentially providing actionable insights in real-world survival problems.

## Introduction

Survival analysis, a fundamental principle in medical research [1], economics, and various other fields, focuses on modeling time-to-event data, where the event of interest might be, for instance, a patient’s recovery [1–3], a mechanical failure [4, 5], or a customer’s churn [6]. This type of data is characterized by the occurrence of a key event or failure over time, along with censored records that are incomplete and thus are not entirely observed [7]. The incomplete observation of data, together with the unpredictability of individual responses to treatment and the multifactorial nature of diseases, presents a particular challenge in survival analysis in medical applications, where the diverse biological characteristics of patients introduce additional complexity.

This complexity is compounded in the prediction of long-term survival, where a primary obstacle is the need for historical data with extended follow-up periods, often more than two decades, which can be difficult to acquire [2]. This requirement inherently limits the inclusion of recent cases whose long-term outcomes have not yet happened, restricting the temporal scope of the dataset, resulting in the exclusion of recent patients treated with modern medical approaches whose outcomes may be most relevant to predictions for new patients. Furthermore, the dynamic nature of risk factors over time, i.e., features relevant to further survival may change as time passes, introduces complexity into the prediction task [8–10]. The time-varying variable effect on survival underscores the importance of individual survival curves, especially in clinical settings, as they allow healthcare providers to understand how a patient’s risk of an event changes throughout the course of treatment or disease progression [11–14]. However, challenges remain in both generating accurate and reliable individual survival curves [14] and quantitatively comparing survival curve performance [15].

To address the challenge of censored data impacting survival prediction via machine learning, we introduce classification-augmented survival estimation (CASE), an integrated approach encompassing dynamic record replication, individual survival curve generation, and exact survival time prediction (the only tool capable of such a prediction, to our knowledge). CASE simplifies the survival prediction by reducing class imbalance typical in survival analysis datasets (far more not-survived than survived records) and by allowing the problem to be represented by simple binary classification rather than by traditional, complex survival models. To support analysis of survival curve predictions, we additionally introduce (1) a novel calibration method, adjusted Bayesian binning-in-quantiles (ABBQ) to directly estimate the survival probability at time *t* for each record; (2) a novel cross-validation method, temporal stratified k-fold cross-validation (TSK-fold), to ensure temporally consistent train/test folds; and (3) two novel survival curve evaluation metrics, individual area under the survival curve (iAUSC) to compare individual curves, and mean AUSC (mAUSC), a dataset-level performance metric.

We demonstrate the effectiveness of CASE on the problem of predicting long-term survival (20 years and longer) in liver transplantation. The empirical study analyzes the implications of CASE for clinical decision making, resource management, and personalized patient care, highlighting the importance of understanding dynamic hazard ratios and the variability of survival probabilities over the entire duration of the study in the context of liver transplantation.

## Related work

Traditionally, long-term survival prediction is examined using established survival analysis techniques, such as Kaplan-Meier (KM) analysis [16] and the Cox proportional hazards model (Cox), also called the Cox model [17]. Although these methods have proven valuable in numerous applications, they face notable limitations when applied to the task of survival analysis in general, particularly in predicting long-term survival [18]. KM curves generate a single survival curve for the entire population without accounting for individual risk profiles and the impact of individual covariates [13, 19]. Cox overcomes some limitations of KM by accounting for the effects of variables on the hazard function, with individual curves obtained by adjusting the baseline hazard function; however, Cox requires the assumption of proportional hazards, which implies that the relative risks associated with different variables remain constant over time for all individuals in the population [3, 20].

Machine learning approaches to generate individual survival curves include random survival forest (RSF) [11], gradient boost survival (GBS) [21], and DeepSurv [22]. RSF struggles with imbalanced survival times [23] which exist in long-term survival prediction. GBS often outperforms Cox and RSF in prediction accuracy but is computationally intensive and provides risk scores that are difficult to translate into survival probabilities [24]. DeepSurv employs deep learning for time-to-event data but inherits proportional hazards limitations and requires large datasets, posing challenges in healthcare settings [22]. DeepSurv additionally does not perform as well as RSF and GBS with tabular data [25, 26], which is generally the most widely available type of data in healthcare applications. Furthermore, these models generally provide variable importance measures averaged over the entire follow-up period, making it difficult to isolate importance at specific time points [27].

In addition to the difficulty in generating survival curves, evaluating survival models and comparing survival curves is a significant challenge due to the lack of standardized methods [15]. Common evaluation metrics like the concordance index (C-index) [28] provide insights into patient risk ordering but does not assess the accuracy of the survival predictions. C-index is also sensitive to the distribution of censored data and may not be suitable for long-term survival predictions [29, 30]. Time-dependent area under the receiver operating characteristic curves (t-AUC) [31] is an extension of the traditional AUC to dynamically evaluate the performance of survival models, but similar to C-index, t-AUC is challenging to calculate with censored data [29, 32] and is also a measure of rank of the data and does not rely on the actual values of the predictions [33]. The integrated Brier score (IBS) [34] assesses probabilistic predictions over time, but is sensitive to model calibration and difficult to interpret due to its squared error component, making it challenging to draw specific conclusions from score differences.

## Materials and methods

CASE restructures the survival problem into a classification task rather than a survival task. In the augmentation step, for each record in the dataset, CASE creates a replicate record for each year of the study duration (augmentation step), and performs classification to predict years survived. By introducing numerous additional records representing survival, CASE mitigates the inherent class imbalance in survival data, allowing for a wider variety of potentially successful classification algorithms to be employed with improved accuracy [35].

**Fig 1:**
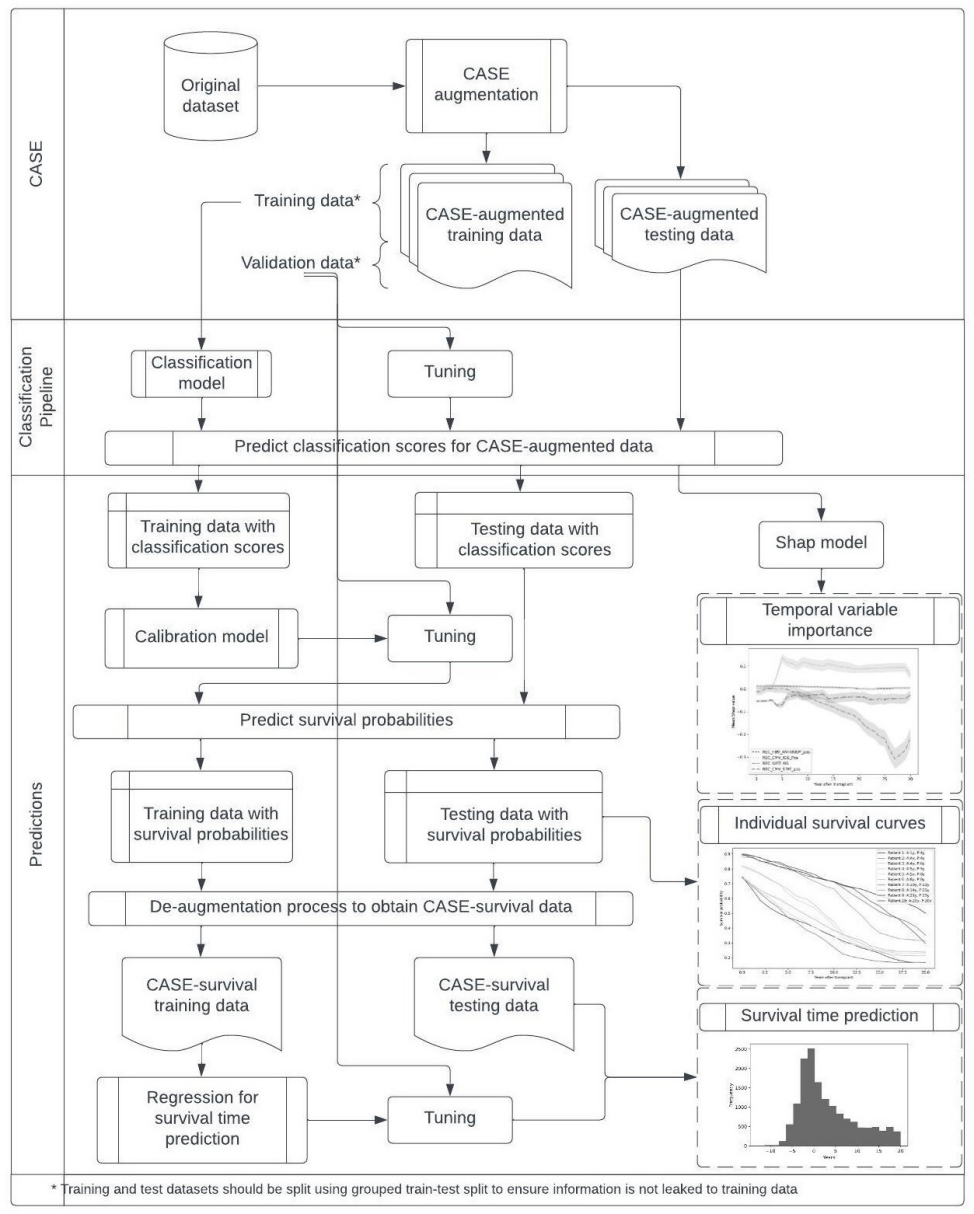
Complete CASE pipeline, from data augmentation to survival curve prediction.

Fig 1 shows the entire CASE pipeline. Initially, the dataset is augmented via the CASE process to create the CASE-augmented dataset, $D_{\text{CASE}}$, which is suitable for classification-based survival prediction. Then, survival probabilities are calculated by calibrating the classification scores, which are later used in the creation of individual survival curves. Next, the calibration step is followed by a de-augmentation (reduction) step where the survival probabilities are added to the original dataset, creating the CASE-survival dataset, $D_{\text{survival}}$. This final dataset is then used to train a regression model to predict survival times and obtain survival curves. The complete code for implementing the CASE framework is available in our GitHub repository (link: https://github.com/hshurabi/case).

Define the original dataset $D=\{\left(x_{i},t_{i},\delta_{i}\right)|i=1,\ldots ,N\}$, where each record *i* consists of (1) a feature vector $x_{i}\in R^{F}$; (2) an observed time to the event or censoring time $t_{i}\in R^{+}$; and (3) a binary event indicator *δ*_*i*_ ∈ {0, 1}, with 1 indicating the occurrence of the event (e.g., death or graft failure) and 0 indicating censoring. Censoring in this context means that no event has yet occurred, but it is important to recognize that absence of an event in the dataset does not mean that the event will not occur, as all records will eventually experience an event (i.e., all patients will eventually die or experience graft failure). If a record *i* is censored, the censoring time *t*_*i*_ is the elapsed number of time periods from initiation of survival analysis (in this case, from the time of liver transplantation) until the current real-world time period. Thus, both *t*_*i*_ and *δ*_*i*_ are required to indicate whether the event actually occurred and when. The objective is to predict the probability of survival for a given time horizon *P*, which is a user-defined parameter that represents the maximum number of time periods of interest after the initiation of the survival analysis to an event (in this case, death or graft failure).

**Fig 2:**
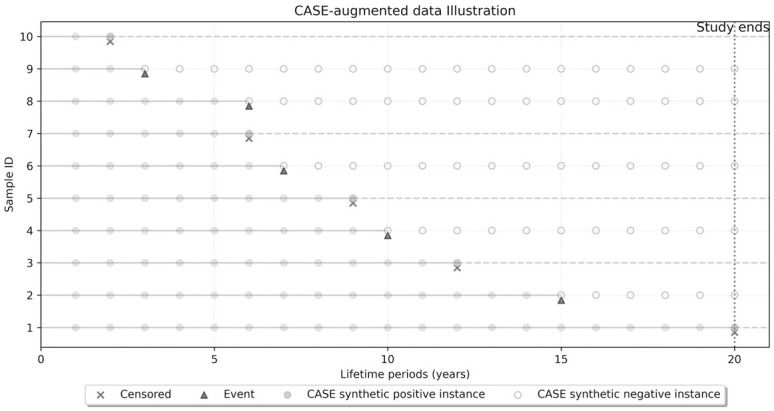
CASE augmentation process with a study period *P* = 20 years.

CASE introduces a transformation operator **T** that increases the size of the dataset by replicating each record up to *P* times (number of periods). This expansion depends on the event and the censoring status, so that an uncensored record is replicated *P* times, each instance corresponding to survival at a distinct period following the study initiation. In contrast, for censored records, replication persists only up to the censoring time *t*_*i*_, thus respecting the bounds of the observed data. This augmentation is illustrated in Fig 2, and the resulting augmented dataset, denoted $D_{\text{CASE}}$, is formally defined as

$$\begin{array}{c} D_{\text{CASE}}=\overset{N}{\underset{i=1}{⋃}}T\left(x_{i},t_{i},\delta_{i}\right) \end{array}$$

where **T**(*x*_*i*_, *t*_*i*_, *δ*_*i*_) yields a set of tuples {(*x*_*i*_, *τ*, *y*_*iτ*_)∣*τ* = 1, …, max(*δ*_*i*_*P*, (1 − *δ*_*i*_) min(*P*, *t*_*i*_))}. *τ* is a new feature added to the augmented record indicating the number of periods elapsed from the initiation of survival analysis. *y*_*iτ*_ is a second new feature (specifically, the target variable) indicating whether the event was observed by time *τ*, and is defined as

$$\begin{array}{c} y_{i\tau }=\{\begin{array}{cc} 1 & \text{if}\;\tau \leq t_{i}\;\text{and}\;\delta_{i}=1\\ 1 & \text{if}\;\tau <t_{i}\;\text{and}\;\delta_{i}=0\\ 0 & \text{otherwise} \end{array} \end{array}$$

For example, if a currently survived patient (*δ*_*i*_ = 0) had a transplant six years ago and we are interested in *P* = 20 years of survival, the censoring time is *t*_*i*_ = 6, and the patient’s record will be replicated six times, ∀*τ* ∈ {1, …6}, with positive records *y*_*iτ*_ = 1, and no replications with *y*_*iτ*_ = 0 (Fig 2, sample ID 7). Conversely, if that patient died (*δ*_*i*_ = 1) *t*_*i*_ = 6 years after transplant (Fig 2, sample ID 8), the record is replicated 20 times with positive records *y*_*iτ*_ = 1 for the first six replications, ∀*τ* ∈ {1, …, 6}, and negative records (*y*_*iτ*_ = 0) for the remaining 14 replications, ∀*τ* ∈ {7, …, 20}.

In Fig 2, the sample size of $D_{\text{CASE}}$ is *n* = 149 with a positive class ratio of 43%, compared to an original sample size of 10 for a survival study, or a sample size of six with a class ratio of 17% for a 20-year binary classification study. Integrating a temporal dimension within the feature space distinguishes each period, allowing CASE to provide a granular survival probability profile throughout the temporal spectrum. This method not only addresses the problem of imbalanced classes commonly found in survival datasets, with up to *N* × *P* additional survival-positive records added, but also utilizes the predictive value of censored data points, which are often underutilized in traditional survival analysis. Introducing a more varied representation of the positive class by incorporating censored data, allows the model to learn from a wider range of survival patterns.

The augmentation process in the CASE framework is designed to replicate real-world clinical follow-up scenarios. For each patient record, new instances are generated for each time period until either the event time or the censoring time. This method reflects how clinicians monitor patient outcomes at regular intervals, capturing the evolution of survival probabilities over time. By creating time-specific records, the approach realistically represents varying survival trajectories, thereby enabling the model to learn temporal risk patterns. Unlike conventional oversampling methods, which repeat the same instances, CASE presents the learning algorithm with more diverse records drawn from real-life observations, avoiding repeating identical patterns.

In CASE, the construction of the target variable as a boolean variable capturing survival is a critical step that enables the transformation of survival analysis into a binary classification framework. By conceptualizing the survival problem as a binary classification rather than a traditional survival prediction, each period *τ* is treated as a separate instance, allowing for discrete survival prediction at that particular point in time. This discrete-time approach contrasts with traditional survival models, which often handle time-to-event data continuously and typically require the proportional hazards assumption. This binary target formulation not only simplifies the predictive modeling task, but also amplifies the dataset’s utility by expanding the number of training instances, particularly for periods where survival data are scarce. Thus, the problem of imbalanced data is effectively addressed by increasing the representation of the survival event at different times, enhancing the robustness of the predictive model.

Training a classification model M on $D_{\text{CASE}}$ allows the use of classification algorithms, avoiding the strict proportional hazards assumptions required by conventional survival models. With each record *i* and the corresponding period *τ*, the model makes a series of binary predictions that indicate whether a record survives or not at each time point. The prediction for survival at time *τ* for a test instance *x*_test_ is obtained by

$$\begin{array}{c} {\hat{y}}_{\text{test},\tau }=M\left(x_{\text{test}},\tau \right),\;\tau =1,\ldots ,P \end{array}$$

where ${\hat{y}}_{\text{test},\tau }$ is a vector of probabilities that describes the survival profile over time. This approach delivers detailed predictions for each interval, capturing the changing risk profile and the dynamic aspect of survival over time.

Note that for test data, where the future is unknown, we propagate each instance through the model *P* times, once for each period, assuming that the status of the instance beyond the current observation point of the study is unknown. This replication enables the model to provide predictions throughout the time horizon, offering a comprehensive view of the survival probabilities at each period. The aggregation of these predictions produces a survival profile over time specific for each instance.

### Class ratio limits

In CASE survival analysis, a crucial factor that influences the balance ratio of the class is the interaction between the distributions of events and censoring instances, along with the selection of the prediction horizon *P*. The CASE approach categorizes dataset records into three distinct types: event cases, survived cases, and censored cases. The creation of new cases in CASE necessarily improves class balance, though how much depends on the distribution of classes in the original dataset, and how events and censoring are spread over the study period, *P*. In this context, class ratio is defined as the ratio of positive cases (positive records) to the total number of records in the dataset. In our liver transplant CASE study, the ratio of survived records improved from 3.66% to 35.15%.

The resulting CASE class balance ratio can be empirically calculated as follows. Define $E_{P}$ and $C_{P}$ as the sets of event and censored records, respectively, for a particular horizon *P* in CASE. Each event record *i* in CASE results in *t*_*i*_ positive cases and *P* − *t*_*i*_ negative cases, while each censored record *i* results in min(*t*_*i*_, *P*) positive cases (Fig 2). Then, the positive class ratio is given by

$$\begin{array}{c} \text{CASE}\;\text{class}\;\text{ratio}=\frac{\sum_{i\in E_{P}}\;t_{i}+\sum_{i\in C_{P}}\;\text{min}\left(t_{i},P\right)}{|D_{\text{CASE}}|} \end{array}$$

As *P* increases, there are fewer censorings and more events, though the impact on both the numerator and denominator in the ratio is dependent on exactly when the censorings and events occur. Thus, while easy to directly calculate, CASE impact on the class ratio is dependent on the specific records present in the original dataset.

High-level observations can be drawn from the CASE class ratio calculation. Consider two extreme scenarios:

1. Minimal censoring: In cases where the survival distribution is skewed such that a majority of events occur in the early periods, censoring is minimal or non-existent. Then, the choice of *P* is less critical. Most records are event cases, leading to a higher representation of the positive class regardless of the length of *P*.
2. Censoring dominance: In contrast, if the dataset is characterized by early censoring, where a significant portion of records are censored in the initial periods, the choice of *P* becomes crucial. A shorter *P* might lead to an under-representation of the positive class, as many records would contribute to the negative class only as cases censored. In such a scenario, a longer *P* can help balance the representation of positive and negative classes.

Thus, generally, if *P* results in minimal censoring, *P* could be lengthened without significant impact on class balance. Conversely, if *P* results in censoring dominance, class balance may improved by increasing *P*.

### Survival probability calibration

To transform the classification scores into calibrated survival probabilities, we introduce a novel adjusted Bayesian binning-in-quantiles (ABBQ) calibration method. The BBQ method is a non-parametric approach to probability calibration, allowing for flexibility in handling complex relationships between raw scores and probabilities [36]. Since the CASE model involves restructuring the survival problem into a classification task and generating probabilities for discrete time points, the BBQ method’s ability to handle non-linear relationships and provide calibrated probabilities based on binning and empirical estimation aligns well with the CASE framework. Our ABBQ method incorporates an “intra-bin variability” term to the BBQ adjustment formula to consider the minor deviations in the raw scores distribution within each bin to capture the variability in the probabilities more effectively.

In the standard BBQ method, raw scores within each bin are transformed based on the empirical survival probability of the bin. However, this approach assumes uniformity of scores within the bin, potentially overlooking minor variations among the records. To address this issue, we introduce the “intra-bin variability” term, which captures the distribution spread of scores within each bin. This term allows for a more granular adjustment of survival probabilities, refining the calibration by accounting for slight deviations in raw scores. As a result, the calibrated probabilities better reflect the inherent uncertainty in survival estimates, leading to more stable and accurate survival curves, especially in imbalanced datasets.

Say classification model M outputs scores *p*_*iτ*_ for each record *i* at time *τ*. First, we sort the scores *p*_*iτ*_ and divide them into *M* bins, *B*_1_, *B*_2_, …, *B*_*M*_, such that each bin contains roughly the same number of scores. The number of bins *M* is a hyperparameter that can be tuned. For each bin *B*_*m*_, we calculate the empirical probability of survival as follows:

$$\begin{array}{c} {\hat{p}}_{\text{emp},m}=\frac{n_{\text{survive},m}}{n_{m}} \end{array}$$

where *n*_survive,*m*_ is the number of survived records in bin *m* and *n*_*m*_ is the total number of records in bin *m*. This empirical probability serves as a reference point for calibrating the raw scores within the bin. Next in the calibration process, we transform the raw scores in each bin to align with the empirical probabilities, effectively adjusting the scores to reflect their true likelihood of survival as follows:

$$\begin{array}{c} {\hat{p}}_{i\tau }={\hat{p}}_{\text{emp},m}+\frac{1}{M}\times \frac{p_{i\tau }-{\text{min}}_{B_{M}}\;p_{i\tau }}{{\text{max}}_{B_{M}}\;p_{i\tau }-{\text{min}}_{B_{M}}\;p_{i\tau }} \end{array}$$

This formula standardizes each bin’s scores into the range [0, 1], preserving the relative differences in survival likelihood among records within the same bin, and then adds the standardized score to the bin’s overall empirical survival probability.

### Individual survival curves

The CASE pipeline introduces a novel method to create individual survival curves *S*_*i*_(*t*), which show the unique risk paths and survival chances for each individual *i* over time *t*. This approach is a departure from traditional survival analysis methods, such as Cox proportional hazards model, which often generate survival curves as variations of a baseline hazard function to indicate the probability that subject *i* survives beyond time *t*. Traditional models assume constant hazard ratios over time for different individuals, meaning that individual risk is just the baseline hazard function, *h*_0_(*t*), adjusted by a set of variables **x**_*i*_ for each individual *i* and their corresponding coefficients ***β***, such that

$$\begin{array}{c} h_{i}\left(t\right)=h_{0}\left(t\right)\times \text{exp}\;(\beta^{⊤}x_{i}) \end{array}$$

where *h*_0_(*t*) is the baseline hazard function estimating the probability of surviving beyond time *t* using non-parametric methods such as the KM estimator, which considers the observed survival times as follows. The survival function in these non-parametric approaches at time *t* is defined as the probability of surviving beyond *t*, given survival up to *t*:

$$\begin{array}{c} S_{i}\left(t\right)=\text{exp}\;(-\int_{0}^{t}h_{i}\left(u\right)du) \end{array}$$

The result is a set of survival curves for different individuals that maintain constant proportional separation, represented as *S*_*i*_(*t*)/*S*_*j*_(*t*) = constant, thus failing to capture the complex interaction of risk factors that can vary significantly throughout the timeline of an instance.

In contrast, CASE focuses on discrete-time instances, allowing dynamic survival predictions not tied to an a priori baseline survival function or to hazards rates that may not be applicable past a certain time. For example, an instance might face a higher risk of adverse events in the first 0–5 years, then enter a stable period. In contrast, another instance might start with a lower risk, but then see a significant increase in risk after 15–20 years. These variations are crucial for understanding and predicting outcomes and are not adequately represented by traditional methods. Moreover, in constructing individual survival curves, censored data are incorporated by limiting the augmentation process to the censoring time for each record ensuring that the survival probabilities are calculated based only on the available observed data. For censored records, the model estimates survival probabilities up to the last observed point, without making assumptions beyond the censoring time.

Within the CASE framework, the survival probability *S*_*i*_(*τ*) at time *τ* is directly estimated as

$$\begin{array}{c} S_{i}\left(\tau \right)={\hat{p}}_{i\tau } \end{array}$$

where ${\hat{p}}_{i\tau }$ is the probability of survival obtained by calibrated classification scores for the survived class (positive class) from the classification model’s output. As CASE provides survival probabilities only for discrete time points, an interpolation method is then used to estimate the survival probability for any non-discrete time *t*:

$$\begin{array}{c} S_{i}\left(t\right)={\hat{p}}_{i\tau_{1}}+\left({\hat{p}}_{i\tau_{2}}-{\hat{p}}_{i\tau_{1}}\right)\times \left(t-\tau_{1}\right) \end{array}$$

where *τ*_1_ = ⌊*t*⌋ and *τ*_2_ = ⌈*t*⌉ are the nearest discrete time points to *t*. Note that *t* − *τ*_1_ is the fractional part of *t*, representing the proportion of time between the lower and upper discrete time points.

### Estimation of survival time

The probability scores ${\hat{p}}_{i\tau }$ create a timeline showing how survival chances change over time, helping to estimate when an event might occur. Define time-to-event, or survival time of a record, as *T*_*i*_. In the original dataset definition, $D=\{\left(x_{i},t_{i},\delta_{i}\right)|i=1,\ldots ,N\}$, for an event, if *δ* = 1, the survival time will be *T*_*i*_ = *t*_*i*_. Note that *T*_*i*_ is unknown for censored cases (*δ* = 0), *T*_*i*_ is unknown, and it is only known that *T*_*i*_ ≥ *t*_*i*_. To determine survival time predictions, we investigate conventional threshold-based estimations, and develop two novel approaches using gradient-based and regression-based estimations.

The simplest method to predict the actual time of an event is to set a threshold *θ* such that if ${\hat{p}}_{i\tau }>\theta$, the instance is considered to survive at period *τ* [37]. The predicted survival time would then be the maximum period *τ* for which ${\hat{p}}_{i\tau }>\theta$. However, thresholds do not consider the unique risk profiles of individuals and how survival probabilities can change over time. It is especially limited when survival probabilities remain relatively stable from year to year, leading to oversimplified and possibly incorrect estimates of when an event might occur.

In our gradient-based approach, we look for the period *τ* with the maximum negative gradient, indicative of a significant decrease in the probability of survival based on the assumption that the steepest decline in survival probability signals the most critical transition period.

In our regression-based approach, we first extend the original dataset with individualized time-dependent survival information by appending each record’s survival probabilities ${\hat{p}}_{i\tau }$ for each period as additional features to the original feature set **x**_*i*_, creating dataset $D_{\text{survival}}$:

$$\begin{array}{c} D_{\text{survival}}=\left[X,{\hat{P}}_{\tau }\right],\;\forall \tau \in \left[1,\ldots ,P\right] \end{array}$$

The regression model R is then trained on $D_{\text{survival}}$ to minimize the difference between predicted survival time ${\hat{T}}_{i}$ and the actual survival time *T*_*i*_ only for $i\in E_{P}$ to ensure accuracy of predictions. In the regression, the predictor variables, which include both survival probabilities and original features, are weighted to minimize the difference in actual v. predicted survival time over the whole population.

### Temporal-stratified k-fold

We introduce temporal-stratified k-fold (TSK-Fold), a novel cross-validation technique specifically designed for survival analysis using the CASE. Traditional k-fold methods randomly select records to be in test/train sets, possibly resulting in some folds having over/underrepresented time intervals in the study period, leading to unrealistic test/train sets and unrealistic model performance assessments for survival curve predictions.

**Fig 3:**
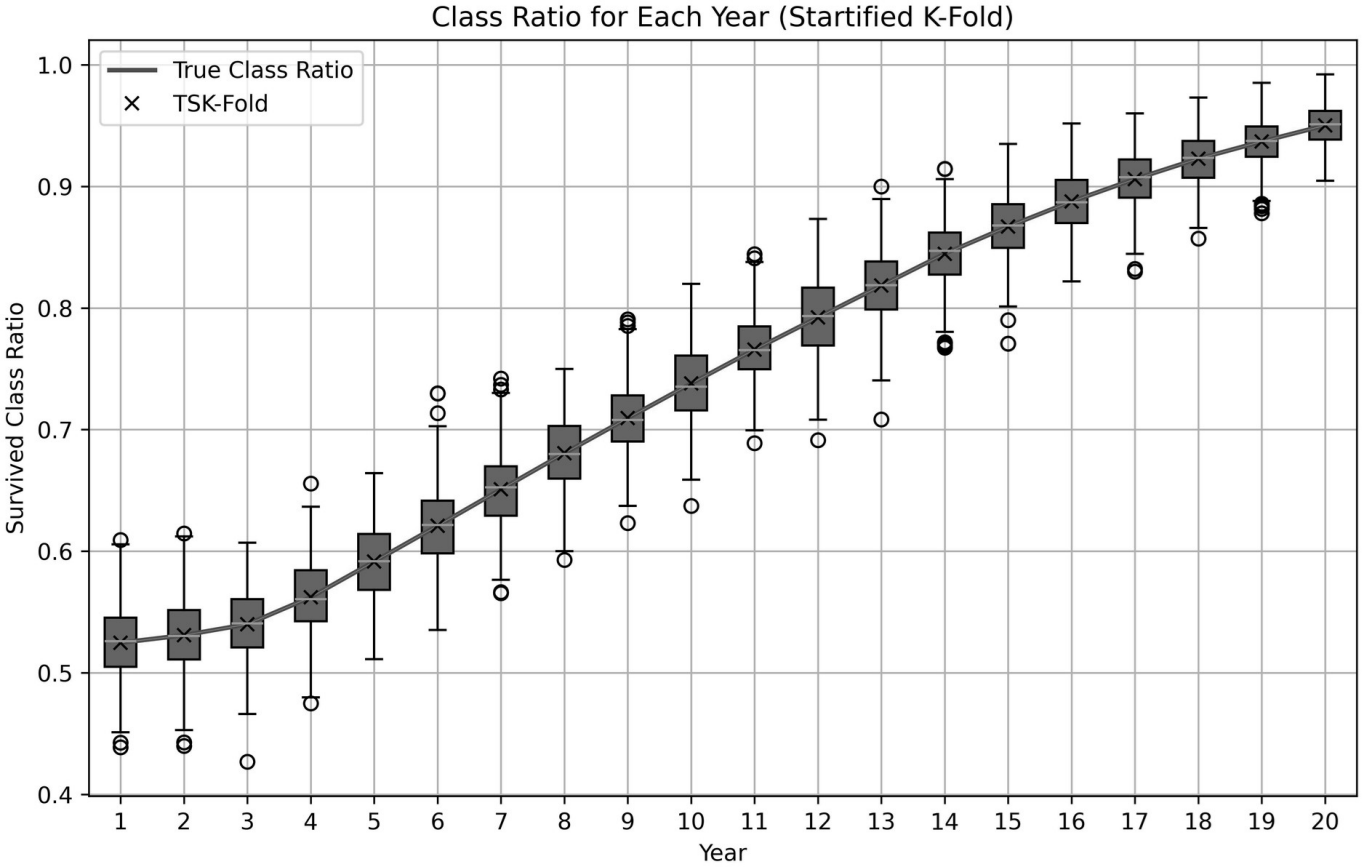
Survived class ratio compared for stratified K-fold and TSK-fold. The plot is generated for 10×10-fold on the $D_{\text{CASE}}$ for liver transplant.

TSK-fold divides the $D_{\text{CASE}}$ into *k* folds, ensuring that each fold contains a representative sample of the entire study period by stratifying the data based on both the time periods and the outcome variable (event occurrence). This dataset is split into time intervals and then stratified within each interval:

$$\begin{array}{c} {\text{Fold}}_{k}=\overset{T}{\underset{t=1}{⋃}}({\text{Positive}}_{t}^{k}\cup {\text{Negative}}_{t}^{k}) \end{array}$$

where ${\text{Positive}}_{t}^{k}$ and ${\text{Negative}}_{t}^{k}$ represent the positive and negative cases for time *t* in fold *k*, respectively. Then, the stratified time intervals are combined to form *K* folds, each of which preserves the temporal structure and maintains class balance (Fig 3). Each fold is used as a validation set once, while the remaining *K* − 1 folds are used for training.

### Individual and mean area under the survival curve

**Fig 4:**
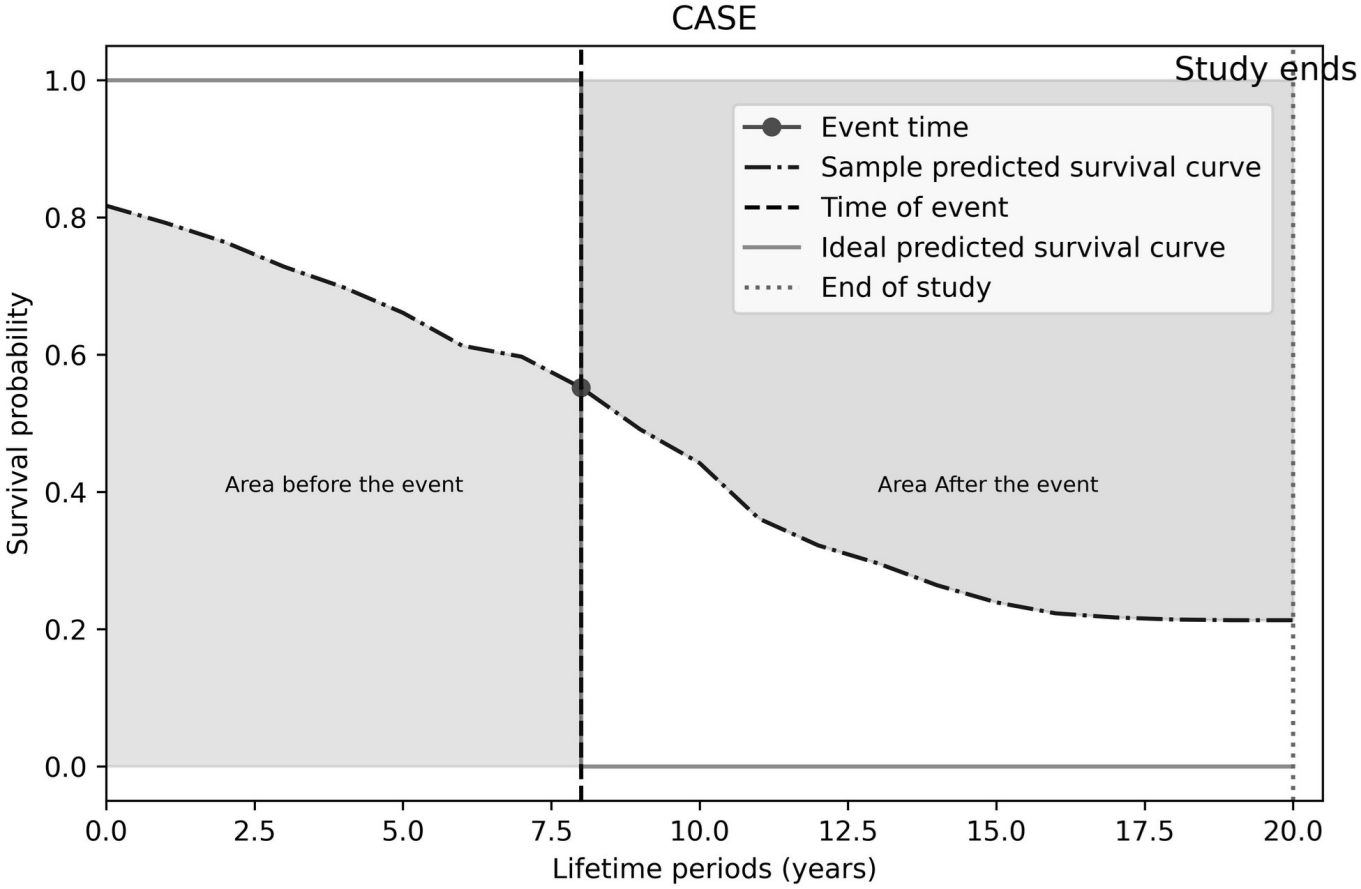
Predicted survival curve derived from an ideal survival model.

To evaluate the accuracy of individual survival curves generated by different models, we introduce a new metric called the individual area under the survival curve (iAUSC), adapted from the widely used Area Under the Receiver Operating Characteristic Curve (AUC) [38] in classification analysis and the Brier score [39], a common metric for assessing the accuracy of probabilistic predictions in survival analysis. iAUSC measures the overall survival probability for each person across the whole study period. In an ideal model, the probability of an observed event is a step function, with 1 for the time leading up to the event, then 0 afterwards. A continuous version of this model follows a sigmoid function (Fig 4):

$$\begin{array}{c} S\left(t\right)=\frac{1}{1+e^{-(M\times \left(t-T_{i}\right))}} \end{array}$$

where *S*(*t*) is the survival function, *T*_*i*_ is the event time for record *i* and acts as a threshold parameter shifting the function on the *x*-axis, and *M* a scaling parameter that indicates the steepness of the curve and the extent of separability between survival outcomes. As *M* approaches ∞, the sigmoid curve exhibits increasingly sharp transitions, resulting in near-perfect discrimination between survival and non-survival events.

To calculate iAUSC for record $i\in E_{P}$, we first determine the weighted area under the survival curve until the event time, and the weighted area above the survival curve after the event time. Unlike the Brier score, which uses squared prediction error, we use absolute prediction error for better interpretability, and emphasize prediction importance around the actual event time with the following weight function:

$$\begin{array}{c} w\left(t\right)=\text{exp}\;(-\frac{1}{P}\left|t-T_{i}\right|) \end{array}$$

where for *T*_*i*_ > *P*, we assume *T*_*i*_ = *P*. The iAUSC for record *i* is then defined as

$$\begin{array}{c} {\text{iAUSC}}_{i}=\frac{\int_{0}^{P}|\hat{S}\left(t|X_{i}\right)-\delta_{i}\left(t\right)|w\left(t\right)dt}{\int_{0}^{P}w\left(t\right)dt} \end{array}$$ (1)

where $\hat{S}\left(t|X_{i}\right)$ represents the predicted survival probability at time *t* given the variables *X*_*i*_. The normalization term in Eq 1 ensures that the iAUSC score appropriately reflects the balance between survival probabilities before and after the event, relative to the overall study duration.

To measure iAUSC performance across the dataset population, we use mean area under the survival curve (mAUSC), the average iAUSC across the set of events in the dataset:

$$\begin{array}{c} \text{mAUSC}\;=\frac{1}{N_{E_{P}}}\sum_{i\in E_{P}}{\text{iAUSC}}_{i} \end{array}$$

Unlike time-dependent AUC [40] or IBS [34], mAUSC does not require period-specific calculations, as iAUSC spans the entire study period for each individual. Additionally, because we focus solely on the accuracy of the model for observed cases, there is no need to weight the scores based on the distribution of censored cases.

### Temporal variable importance

To analyze these time-varying effects of variables, we leverage SHAP (SHapley Additive exPlanations) [9] values within the CASE pipeline. SHAP values quantify the contribution of each feature to the model’s prediction for each record, adapted to CASE augmentation by defining the SHAP value *ϕ*_*i*,*j*_(*τ*) for feature *j* in instance (*i*, *τ*) as the effect of feature *j* on the prediction for instance *i* at time *τ*. The importance of feature *j* at time *τ* is then just the average of these SHAP values for all records that survived *τ* periods:

$$\begin{array}{c} {\bar{\phi }}_{j,\tau }=\frac{1}{|I_{\tau }|}\sum_{i\in I_{\tau }}\phi_{i,j}\left(\tau \right) \end{array}$$

where $I_{\tau }$ is the set of instances survived at time *τ*.

These time-varying SHAP values can provide population-level insights into important features, and individual-level insights can be obtained by examining a single record’s time-varying SHAP values.

## Case study: Long-term graft survival in liver transplant patients

Orthotopic liver transplantation is a critical intervention for end-stage liver disease patients, offering a renewed opportunity for extended survival and improved quality of life [41]. Although the field has seen many machine learning studies focusing on short- to mid-term post-transplant outcomes (see, e.g., the systematic review [42]), only one study examined survival longer than 10 years [43] and few studies used exclusively pre-transplant information [43–46].

We apply CASE to the task of predicting long-term, specifically 20-year, graft survival in liver transplant patients using only pre-transplant information. The lengthy survival period creates challenges in that only patients who received transplants 30+ years ago have known (non-censored) graft survival, these account for 3.66% of all patients. Our objective is to generate predictive insights that could be used at the time of transplantation, helping physicians understand the survival probabilities of patient-donor matches where multiple donors may be available and the overall patient survival profile before surgery.

Data was compiled from the publicly available Scientific Registry of Transplant Recipients (SRTR) in the United States [47], also called the UNOS/OPTN dataset, which contains records of liver transplant recipients from 1987 to 2021. We applied the following pre-processing criteria for SRTR records to be included in our dataset. We limited the patient records to those with transplants in February 14, 2016 or earlier, ensuring at least a five-year follow-up period. We excluded pediatric cases (age ≤ 18 years), instances of multi-organ transplants, and variables related to perioperative and post-transplantation periods or that had more than 80% missing data. The only exception was the split liver variable, which was included due to its clinical significance, despite having more than 80% missing. A mean imputation strategy was used to fill in missing data in the remaining variables, as more sophisticated algorithms often do not improve the predictive performance of machine learning models applied in healthcare data [48].

**Fig 5:**
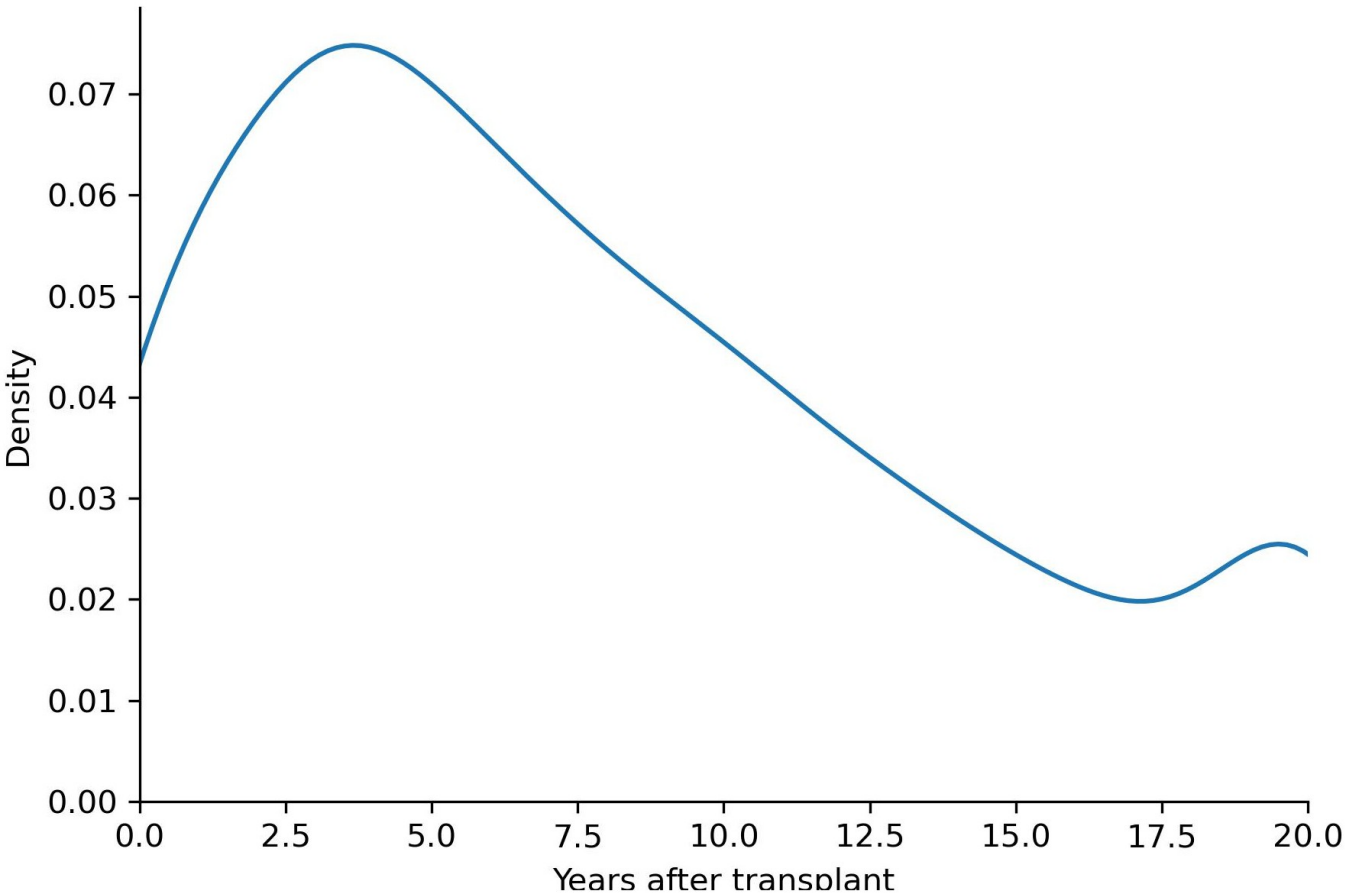
Distribution of observed survival times in the liver transplant SRTR dataset.

The final dataset D included 118,419 records and 107 pre-transplant variables (26 numerical, 81 categorical). Among these variables, 36 were donor-specific, while 71 were recipient-specific. The details of these variables, along with their definitions and information regarding missing data, are available in S1 Table. The 5-, 20-, and 30-year graft survival percentages (class ratios) are 66.63%, 14.82%, and 3.66%, respectively. After implementing the CASE model, the augmented dataset included 2,568,202 records with a 35.15% class ratio. The survival distribution of the final dataset is shown in Fig 5. Note that there is an increase in probability of long-term survival after about 17.5 years post-transplant, indicating that patients who survived 17.5 years are more likely to survive to about 20 years. This observation demonstrates that common monotonically decreasing survival curves may not provide the most accurate survival curve shape for all datasets.

## Results

We first evaluate CASE’s ability to predict long-term survival at 20-, 25-, and 30-years post-transplant via classification, testing both Random Forest (RF) [49] and XGBoost (XGB) [50] models, both of which have had widespread adoption and successful applications in numerous studies [25, 51]. Bayesian optimization [52] was employed for hyperparameter tuning (S1 Appendix).

We then evaluate CASE’s survival model performance in comparison to common survival curve models—RSF, Cox, and KM—and examine temporal variable importance.

### *t*-year survival prediction

To demonstrate the effectiveness of CASE augmentation, RF and XGB models are trained on $D_{\text{CASE}}$ and also on D for classification of 20-, 25-, and 30-year survival post-transplant. Both CASE-augmented models produced notable improvements in performance (Table 1), with 27% AUC and 56% F1 score improvement. Additionally, the Matthews correlation coefficient (MCC) [53, 54] showed significant improvements, 69% with RF, and 65% on XGB. It is important to note that in the context of a CASE-augmented model, the AUC reflects the model’s performance in distinguishing between the survived and non-survived classes across the entire time spectrum, as opposed to a binary classification model’s AUC that predicts the class at a single time period *t*, and thus is only a measure of model performance at time *t* and not over a full study period.

**Table 1:** 20-, 25-, and 30-year graft survival prediction performance. Bold indicates best performance.

Model	Metric	D			$D_{\text{CASE}}$
		20-year	25-year	30-year	
RF	AUC	0.69	0.71	0.73	**0.87**
	F1 Score	0.32	0.24	0.20	**0.73**
	MCC	0.18	0.14	0.12	**0.58**
XGB	AUC	0.69	0.71	0.73	**0.88**
	F1 Score	0.32	0.25	0.21	**0.73**
	MCC	0.19	0.16	0.20	**0.57**

As the survival year to be predicted increases from 20 to 25 to 30 years, AUC predictions increase in the standard models, which could be misinterpreted as indicating more accurate or simpler predictions for longer-term survival. However, a higher AUC in this context more likely means that the model guesses the majority class correctly for most instances while failing to correctly identify minority class instances due to the increasing class imbalance [55]. The F1 and MCC scores likely provide a more accurate understanding of model performance, as they provide a balance between precision and recall, especially important in imbalanced datasets [56].

Given the similar performance of the RF and XGB models (Table 1) and the fact that both models are tree-based, we proceed with XGB for subsequent analysis. XGB holds an additional advantage over RF in the context of variable importance analysis using SHAP values, shown to yield actionable clinical insights in a bone marrow transplant survival prediction [57], though such analysis is out of scope here.

### ABBQ calibration

**Fig 6:**
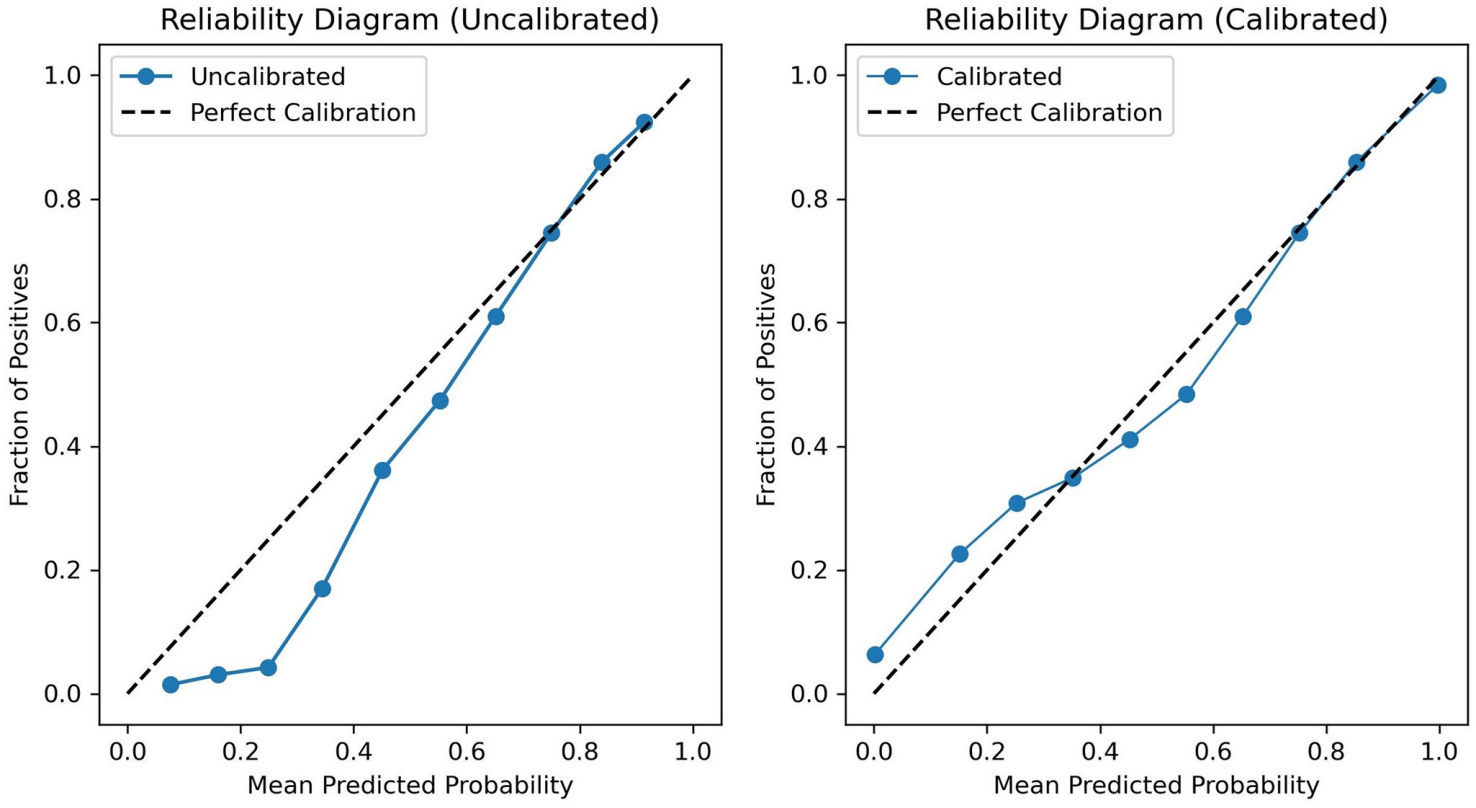
LT: Reliability diagram for ABBQ calibration.

To find the optimal value of hyperparameter *M* in the ABBQ method, we employed Bayesian optimization. We tested values of *M* within a predefined range (e.g., 5 to 100) and selected the value that maximized calibration performance based on the validation set. This approach ensured that the chosen *M* provided the best balance between calibration accuracy and model stability. Fig 6 shows the reliability diagrams comparing the calibrated and uncalibrated models. For the uncalibrated model, we observe that predicted probabilities tend to underestimate the actual event rates for lower probability predictions, whereas, for higher probability predictions, the uncalibrated model is almost perfectly aligned with the ideal calibration line.

After calibration, the predicted probabilities aligned better with the observed outcomes, particularly in the low and mid-range probability regions. The expected calibration error (ECE) for the uncalibrated model was 0.1117, whereas, after calibration, the ECE decreased to 0.1061, indicating an improvement in how well the predicted probabilities reflect the true event likelihood.

### Survival curve model performance

The performance of CASE, RSF, Cox, and KM survival models was examined with C-index [28], IBS [34], t-AUC [40], and mAUSC (Table 2). Note that KM offers a single survival function estimation for the entire population, so it has no C-index calculation. CASE demonstrated superior performance on all metrics, though all metrics ranked the models in the same order (CASE, Cox, RSF, then KM), with the exception of mAUSC, which ranked KM slightly ahead of Cox.

**Table 2:** Survival model performance. Bold indicates best performance.

Model	C-index	IBS	t-AUSC	mAUSC
KM	N/A	0.3182	0.50	0.60
Cox	0.53	0.3138	0.55	0.59
RSF	0.51	0.3167	0.52	0.56
CASE	**0.58**	**0.2899**	**0.60**	**0.62**

**Fig 7:**
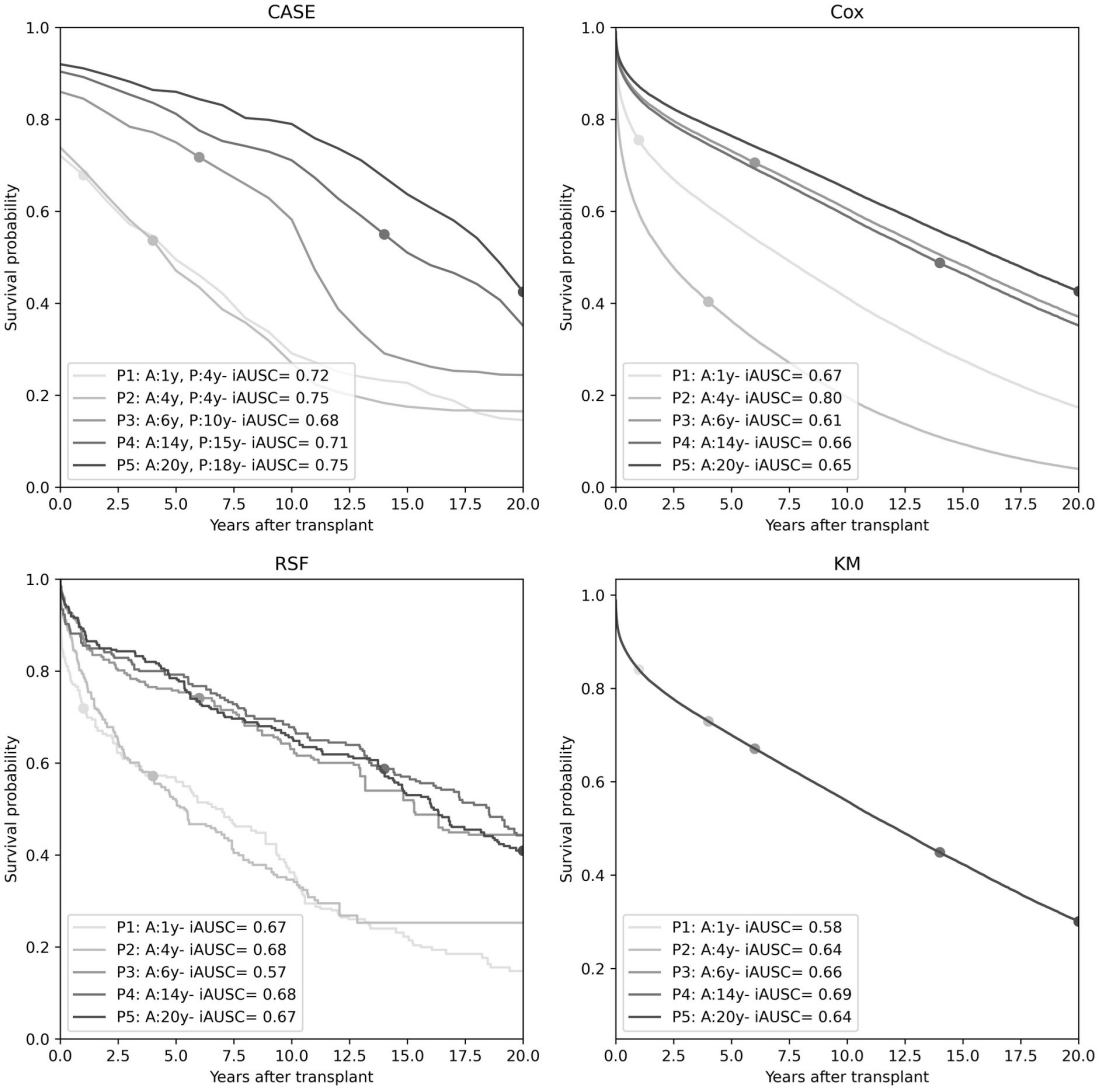
Survival curves for five randomly selected patients with actual time marked. Note that KM produces only a single population curve. A: actual graft survival; P: predicted graft survival.

The survival lines in Fig 7 show survival curve predictions for five randomly selected patients, numbered in order of increasing actual survival. Patients 1–3 are the shortest-surviving, with survivals of 1–6 years, while patients 4 and 5 survived 15–18 years. Interestingly, CASE, Cox, and RSF all show curve separation of the shortest-surviving and longest-surviving patients. However, Cox and RSF curves for long-surviving patients are very similar, indicating that while these models can distinguish between short and long survival times, they may fail to capture the differences between varying medium- and long-term survivals (e.g., 6, 14, and 20 years). CASE is the only model able to differentiate the medium-surviving patient 3, but it is important to note that only a small sample of individual curves are analyzed here. Additionally, all the models poorly capture patient 3’s actual graft survival, and the iAUSC of this patient is correspondingly the lowest of the five patients for all models except KM, which generally has worse iAUSC for these patients than the other models.

For patient 5, the CASE curve begins with a high survival probability but shows a gradual decline over time, notably more gradual than for the other patients. This pattern could reflect a patient whose post-transplant condition is declining at a predictable rate, allowing timely clinical interventions. In contrast, patient 3 initially follows a similar trajectory with a high survival probability, but then shows a sharper decline around year 11.

In contrast, the Cox curves follow a consistent pattern of proportional hazards, a direct consequence of the model’s underlying assumption. In some cases, such as patients 1 and 2, the Cox model provides inaccurate relative curves, where patients who survived longer have higher risk than those who survived for shorter time, although all the models experience some level of this discrepancy according to the C-index values in Table 2.

**Fig 8:**
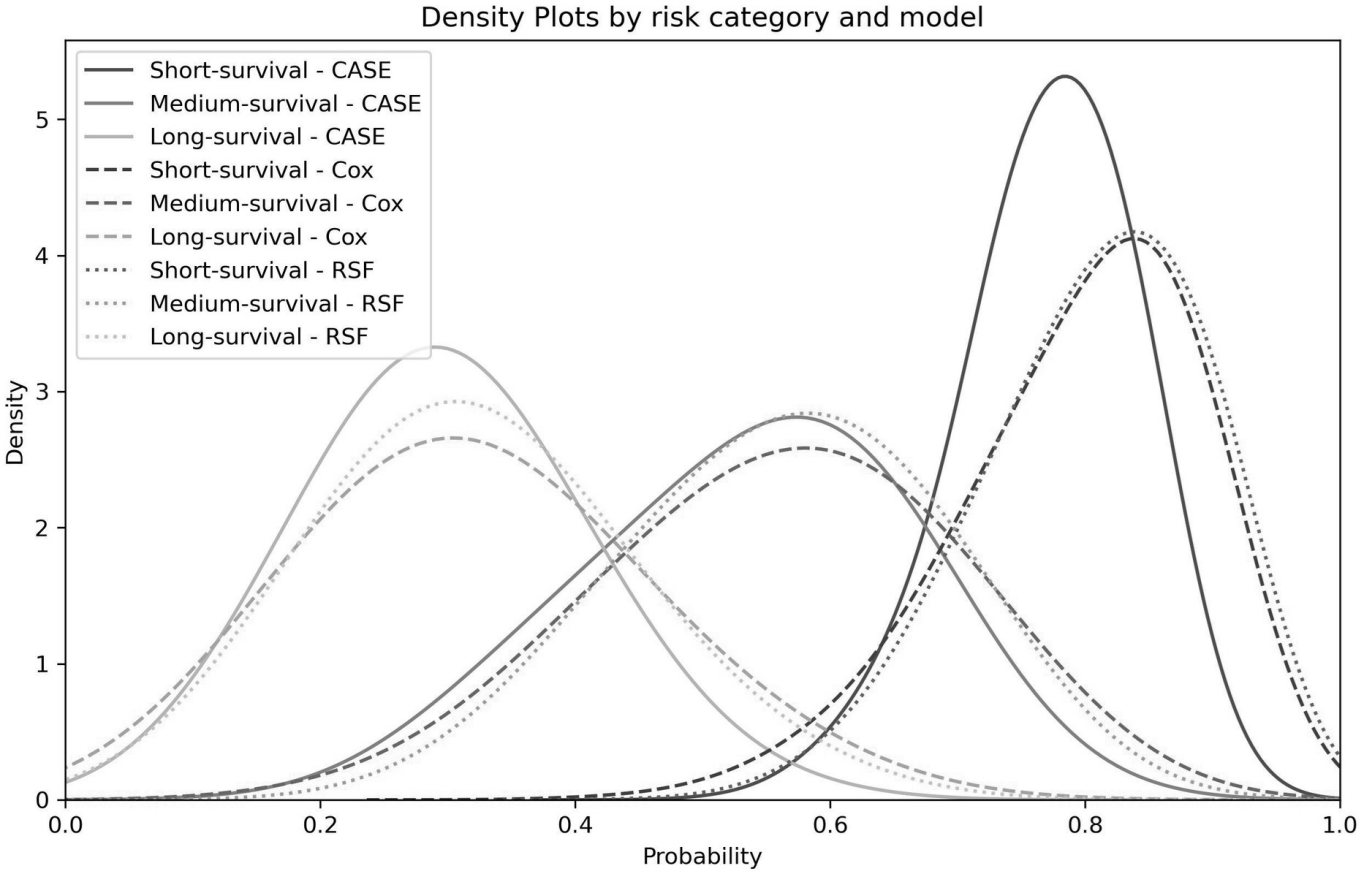
Distribution of predicted survival probabilities at the time of event.

We additionally analyzed the predicted probabilities of survival at the time of the event through density plots of predicted survival probability at time of event for short (<5 years), medium (5–15 years), and long survival (>15 years) patients (Fig 8). Better-performing models should have distributions shifted to the left, indicating a lower, more accurate probability of survival at the time of the event. CASE noticeably outperforms Cox and RSF in this regard for short and especially long survival patients, and all three models are similar for medium survivals, though the CASE plot falls off more sharply, which is preferred.

### Point-estimation of survival time

**Fig 9:**
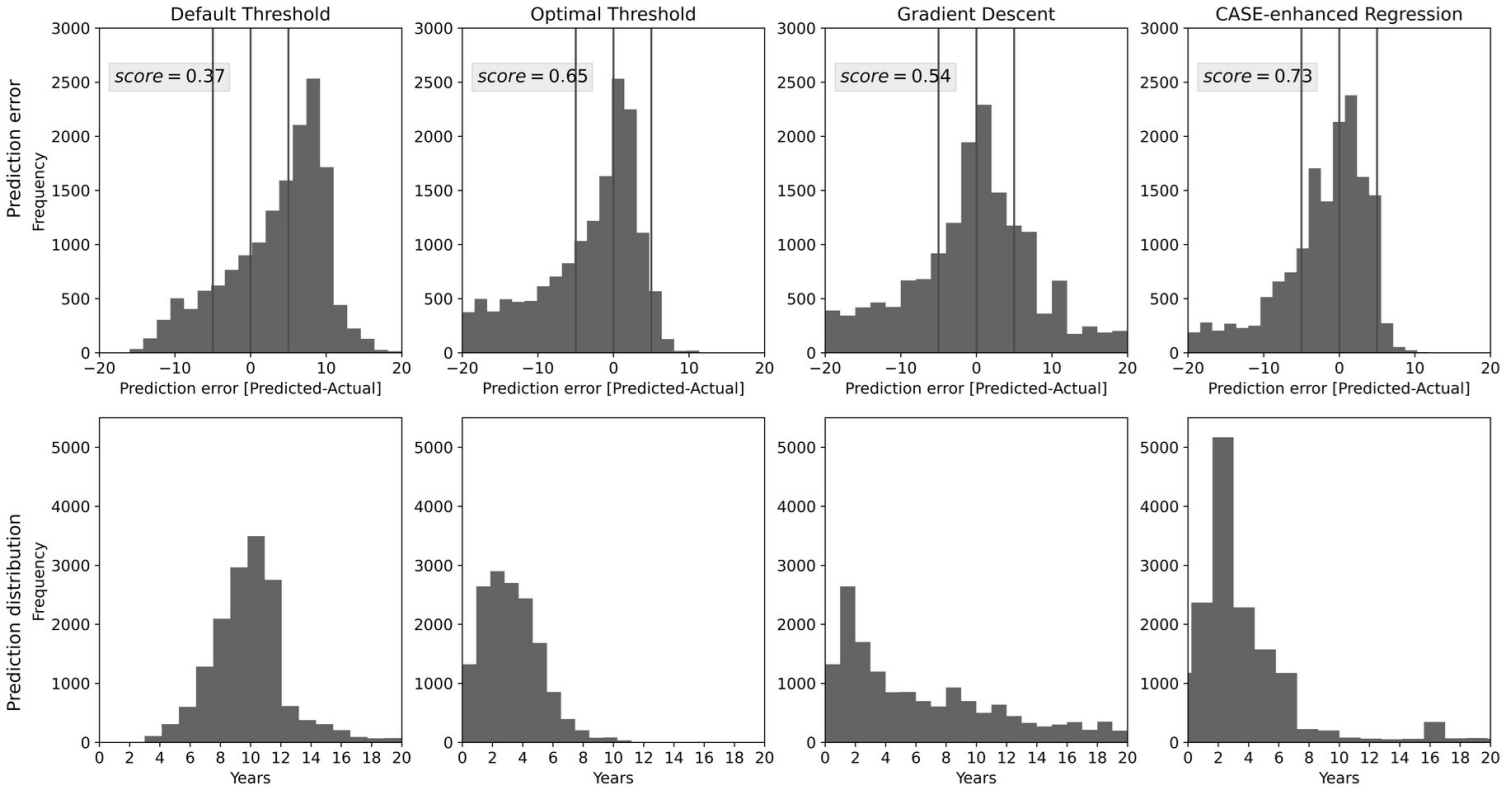
CASE difference between actual and predicted survival times. Top: Prediction error, with negative values indicating an overestimation of survival times by the model and positive values indicating an underestimation. Bottom: Prediction distribution.

To our knowledge, CASE is the only survival model able to produce a point estimate of survival time, that is, predictions of exact survival time. We evaluate the accuracy of CASE’s point-estimate survival predictions using threshold-based, gradient-based, and regression-based estimations, specifically predicting a five-year window of graft survival (Fig 9). As expected, the regression-based method significantly outperforms the other approaches with 73% prediction accuracy, defined as the percent of patients with a survival time correctly predicted within a five-year window.

### Temporal variable importance

**Fig 10:**
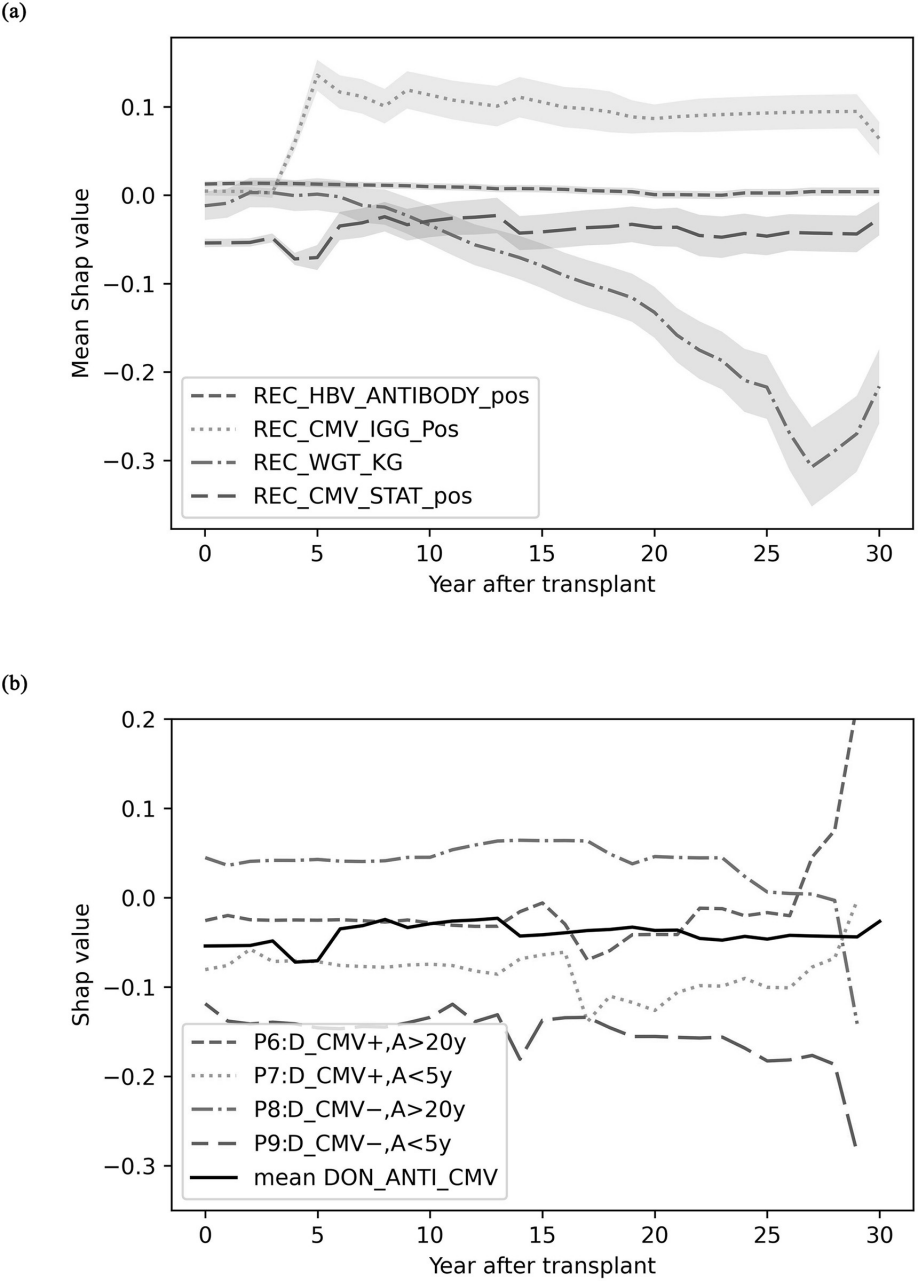
Temporal variable importance analysis. A: Actual survival time, D_CMV+/−: positive/negative donor anti CMV status. (a) Selected recipient features (b) Donor CMV for selected patients.

The temporal variable importance in Fig 10a shows how the significance of different variables changes over time using four recipient variables as examples: REC_HBV_ANTIBODY_pos (HBV antibody positive), REC_CMV_IGG_Pos (CMV IgG positive), REC_WGT_KG (weight), and REC_CMV_STAT_pos (CMV status at time of transplant). The presence of HBV antibodies in the patient has a nearly constant near-zero impact on survival throughout the post-transplant study period, while CMV status has slightly less stable but fairly low negative impact on survival. Interestingly, CMV IgG positive status has almost no importance for the first four years, after which it becomes a strong positive indicator for survival. The recipient’s weight has a steadily increasing negative influence on survival up to around 27 years post-transplant, possibly due to its association with other long-term health conditions, after which it decreases in importance, but is still negatively associated with survival.

To analyze how individual variable importance might differ from the overall trend, Fig 10b shows the SHAP importance for the donor’s CMV antibody status (DON_ANTI_CMV) over time for four selected patients: CMV+ and CMV− for short (< 5 years) and long (> 20 years) survival. The overall mean SHAP value for DON_ANTI_CMV remains relatively stable over time, but the individual trends for the four patients vary significantly from the mean curve, indicating that not only do feature importances change over time, but they may have different impacts on different patients. While both patients with a CMV− donor (patients 8 and 9) exhibit a similar trend of steady influence until about 28 years post-transplant, the feature has a positive impact on patient 8 (who had a long survival) and a negative impact on patient 8 (who had a short survival). A similar pattern can be observed for patients 6 and 7, who had CMV+ donors. These observations may indicate that although donor CMV status seems relatively unimportant when looking at mean importance, it may be a useful individual indicator of short survival, and an indicator for very long survival (>25 years).

## Discussion

To our knowledge, the proposed CASE approach is the first to transform survival analysis into a classification task using machine learning techniques, thereby enhancing predictive accuracy and managing complex, non-linear relationships. It is additionally the only survival model capable of providing exact survival time estimates, to our knowledge, and yielded 73% prediction accuracy on the liver transplant long-term survival case study. The data augmentation in CASE is an alternative to oversampling to improves class imbalance, and resulted in improved prediction performance compared to KM, Cox, and RSF in the case study. While it is common to utilize oversampling techniques to overcome class imbalance [58], oversampling often fails when put to real-world problems since the synthesized samples may not truly belong to the minority class [59].

The visibly different individual survival curves generated by CASE compared to the single population KM curve or the same-trajectory individual Cox curves support previous findings that KM and Cox may fail to identify time-varying covariates and capture the complexities of survival data, especially for long-term survival [10, 19, 60, 61]. CASE’s approach to generate individual survival curves by the ABBQ method ensures robust calibration of predicted survival probabilities, reflecting true survival chances more accurately according to the examined metrics. Our TSK-fold cross-validation method ensures that the temporal structure of the data is preserved while maintaining balanced classes within each fold, unlike traditional *k*-fold methods that randomize this temporal structure, leading to unrealistic training and validation sets [62]. In survival analysis, preserving the temporal order of events is crucial because the risk of events and covariates may change over time [10, 63], e.g., as medical standards and technologies change through the years. CASE’s ability to capture these risks and provide improved survival curves is indicated by its performance in the liver case study, where RF and XGB classifier AUCs improved from 0.69–0.73 to 0.87–0.88 (mean 0.16 improvement) and F1 scores from 0.20–0.32 to 0.73 (mean 0.61 improvement); similarly, survival model C-index and t-AUC metrics improved by an average of 0.06 and 0.08, respectively, while IBS and mAUSC showed more modest average improvements of 0.03 and 0.04, respectively.

While there are statistical studies to predict liver transplant survival (e.g., [64–66]), we focus our discussion on machine learning methods, which include RF [67], RSF [68], logistic regression [69], Cox regression [70], artificial neural networks [43, 67, 68, 71–73], Bayesian networks [44], deep learning [69], PSSP [45], and even unsupervised and semi-supervised methods [46, 74]. Many of these studies only considered survival times of three months or less [44, 67, 71, 72], while a 13-year prediction time [43] is the only study period greater than 10 years. Despite the short- and medium-term survival predictions, most AUCs were ≈0.56–0.73, and few studies obtained AUCs over 0.85 [43, 73]. The differences in study periods and datasets make direct comparison to CASE performance difficult, but the fact that only one study [43] exceeded our 0.88 AUC (only for some years in the 1–10 year survival range) despite our more challenging >20-year prediction indicates that the CASE framework is likely an improvement over conventional machine learning methods. For a more direct comparison, of the three studies that used the same SRTR dataset as our case study (though in different years) [43–45, 68–70] and exclusively pre-transplant variables [43–45], one only examined three-month survival with an AUC of 0.64 [44] and another did not provide any conventional performance metrics for their 10-year survival prediction [45]. The most recent of these studies [43] used a highly curated patient set (383 patients of the available 65 535), which may explain their very unusual oscillating AUCs in the range ≈0.85–0.99 for one- to 10-year predictions, after which AUCs fell sharply to ≈0.45 at 13 years. It is therefore reasonable to conclude that CASE outperforms other machine learning methods for survival prediction, at least in the liver transplant context, even with a significantly longer study period and exclusion of post-transplant information. In particular, our exclusion of post-transplant variables while maintaining high accuracy allows clinicians to make more individualized patient decisions before transplant, possibly guiding the patient-donor matching process.

To the best of our knowledge, there is no established score for evaluating the accuracy of the survival curves [15]. In the survival literature, visually identifying the curve that outperforms others is the primary method [75]. While the log-rank test is commonly used for comparing KM curves, its power diminishes in cases of non-proportional hazards [76]. C-index is a measure of the rank of the data and does not rely on the actual values of the predictions. It is also highly sensitive to the distribution of censored cases and is usually upward biased [29], and therefore may not be suitable for evaluating long-term survival or *t*-year survival probabilities [30]. t-AUC is also rank-based, with the same limitations as C-index [29, 32, 33]. The novel mAUSC metric we introduced attempts to address the limitations of inflated C-index and t-AUC scores, a common challenge in long-term survival, by incorporating time-weighted considerations, which may make mAUSC more reliable under varying study periods. Interestingly, the existing C-index, IBS, and t-AUSC metrics all rank the models in the same order (CASE, Cox, RSF, then KM), while mAUSC differs in that it ranks KM as the second-best model. The individual iAUSC metrics used to calculate overall mAUSC may also provide insight into how much confidence a clinician should place on one particular patient’s predicted survival curve.

Our novel SHAP-based temporal variable importance curves provide a continuum of individualized variable importance over time, unlike other previous attempts to evaluate time-varying importance through successive hazard ratios [10, 77], simple statistical analysis [63], or one-off classifications for different survival times to illustrate differences in variable importances [78], which is not the same as variable importances dynamically changing over time. Additionally, these previous approaches examined population risk changes, not individual changes. Our approach may provide more accurate lifetime information for clinicians, and indicate future periods during which interventions may be appropriate for individual patients. We found some similar trends to mortality in kidney dialysis in that patient weight is less significant for early survival but becomes increasingly important for long-term survival [10].

While CASE represents a significant advancement in survival analysis by improving long-term survival predictions and personalized patient care, it is not without limitations. One major issue is the impact of censoring and event distributions on CASE’s augmentation process. The choice of study period *P* is crucial; we recommend choosing a *P* less than the maximum survival time in the dataset, ensuring that most of the survived cases are captured.

While the ABBQ method enhances the calibration of survival probabilities, it has potential limitations. The method generally performs best when there is a sufficient amount of data within each bin, as too few records can reduce the reliability of the calibrated estimates. Additionally, ABBQ may be sensitive to highly skewed data distributions, where extreme values or imbalanced class ratios could impact the binning process and overall calibration accuracy. In addition, while temporal stratification in TSK-Fold ensures a more realistic evaluation of survival models, it can face challenges if certain time intervals contain very few events. To address this issue, we select time intervals and the number of folds (*k*) based on event density, aiming to balance representation across folds. While TSK-Fold is primarily beneficial for medical applications, it can also be adapted for financial risk analysis, engineering reliability, and customer churn prediction, where time-dependent patterns are crucial. However, alternative validation methods such as walk-forward validation [79] may offer more flexibility depending on the dataset.

While metrics like the C-index [28] and time-dependent AUC [40] primarily assess the ranking accuracy of predicted survival probabilities, they do not directly evaluate the calibration of survival curves over time. The Brier score [34] measures the squared error between predicted and actual outcomes but does not offer clear interpretability for individual predictions. In contrast, iAUSC and mAUSC provide a more direct evaluation of survival probability accuracy at each time point, making them more suitable for applications where individual survival estimation is critical. These metrics offer complementary insights to traditional measures, with iAUSC focusing on individual-level accuracy and mAUSC capturing population-level performance. Despite the advantages of mAUSC, it only assesses model performance for observed cases, making it less effective for datasets with a low incidence of events. It is important to acknowledge that in extremely imbalanced datasets, any performance metric may not fully reflect the model’s accuracy [29, 32]. Another limitation of our framework is the computational intensity of calculating SHAP values for temporal variable importance, especially given that the CASE-augmented dataset will be much larger than the original dataset.

Finally, CASE was tested on a single US national dataset (SRTR), and should be trained and tested on a hospital’s own past patient data prior to implementation. More testing on a variety of survival-oriented datasets is necessary to understand CASE’s generalizability, though the improved class imbalance alone should provide improved prediction performance. We only tested CASE using RF and XGB classifiers, and further testing with other classifiers could prove interesting, though may impact feature interpretability since RF and XGB lend themselves to human-understandable feature importance.

## Conclusion

We introduced the CASE framework, which offers an innovative approach to survival analysis by transforming it into a classification task. CASE integrates effectively with established machine learning algorithms, providing a practical solution for handling censored data. With its unique augmentation process, CASE addresses the dataset imbalance issue, which is common in most survival analysis studies, especially in long-term survival prediction. The ability to generate accurate individual survival curves sets CASE apart from traditional methods like Kaplan-Meier and Cox models, offering a more detailed and personalized understanding of patient outcomes. Additionally, the novel regression method within the CASE framework offers direct prediction of survival times, further enhancing its utility. Clinicians can use the insights provided by CASE to move beyond binary paradigm of survival predictions toward a more holistic approach that considers survival probabilities over time. However, it is essential to interpret these findings with caution. Although model predictions can be valuable for identifying periods of increased risk, actual clinical outcomes may depend on many factors, including interventions taken, changes in patient health or behavior, or advancements in medical care throughout the patient’s post-transplant life.

Additionally, CASE generates temporal variable importance curves using SHAP values, which evaluate the impact of variables on survival over time, aiding in the development of personalized treatment strategies. The introduction of the iAUSC and mAUSC metrics provide new tools for evaluating the accuracy of survival predictions for individuals and for whole datasets. The CASE framework yielded improved survival curve accuracy across all tested metrics—C-index, IBS, t-AUC, mAUSC—and additionally significantly improved AUC and F1 scores in classification survival methods. These more accurate survival predictions and facilitate the patient-donor matching process. Its ability to forecast critical health events within a time frame that is significant for clinical decision making may lead to better individualized health care strategies and improved patient outcomes.

Future work will focus on improving the practical application of CASE, including optimizing the computational efficiency of SHAP value calculations and testing the generalizability of CASE across different datasets, particularly small datasets and those with high proportions of censored data, as well as exploring the application of additional classification models to assess the robustness of the method. We also encourage future work to explore different values of *P* across various diseases and datasets to identify the optimal prediction horizon for specific applications, where no medical constraints on the study period exist and sufficient follow-up data is available. In addition, the broader adoption of iAUSC and mAUSC metrics requires validation and consensus within the research and clinical communities, as well as comparative studies to establish their advantages over traditional survival metrics. It is worth mentioning that although our case study and analysis focus on a healthcare survival problem, CASE is broadly applicable to any survival prediction problem, including equipment reliability, finance, and customer relationship management.

## Supporting information

S1 Table Variable details. (TEX)

S1 Appendix Hyperparameter tuning details. (PDF)
