# Supplementary material for: Classification-augmented survival estimation (CASE): A novel method for individualized long-term survival prediction with application to liver transplantation
Source: PLoS One. 2025 Jan 17;20(1):e0315928. doi: 10.1371/journal.pone.0315928 (PMC11741629; doi:10.1371/journal.pone.0315928)
Supplement: S1 Appendix — (PDF) [file pone.0315928.s002.pdf]

S2 Appendix. Hyper-parameter Tuning Details

We performed Bayesian optimization algorithm to tune the hyper-parameters of the RF, RSF, and XGB models.

The hyper-parameters that were tuned for the RF and RSF models included:

- Minimum samples split: The minimum number of samples required to split an internal node. We used the range $(2, 50)$.
- Maximum depth: The maximum depth of the tree. We used the range $(2, 50)$.
- Minimum samples leaf: The minimum number of samples required to be at a leaf node. We used the range $(1, 10)$.
- Maximum leaf nodes: Limits the maximum number of leaf nodes in the decision tree. We used the range $(20, 100)$.
- Maximum features: Sets the maximum number of features used in each individual decision tree. We used both ranges and predefined functions $(10, 70)$.
- Number of estimators: Sets the number of decision trees to be used in the ensemble model. We used the range $(100, 1500)$.

The hyper-parameters that were tuned for the XGB models included:

- Learning rate: learning rate of the optimization algorithm. We used the range (0, 1) as the search space.
- Maximum depth: maximum depth of the tree. We used the range (1, 200).
- Minimum child weight: The minimum sum of instance weights needed in a child node of a decision tree and helps to control overfitting by adding a constraint on the minimum number of samples required in each child gamma. We used the range (0, 10).
- gamma: The minimum loss reduction required to make a further partition on a leaf node of a decision tree and helps to control the complexity and smoothness of the decision boundary by adding a regularization term to the objective function. We use the range (0, 100).
- Sub-sample: Determines the fraction of observations (samples) to be randomly sampled and used in the construction of each tree in the ensemble. We used the range (0.01, 1).
- Column sample by tree: The fraction of features (columns) to be randomly sampled and used in the construction of each tree in the ensemble and helps to increase diversity and reduce correlation among the trees in the ensemble. We used the range (0.01, 1).
- Column sample by level: The fraction of features (columns) to be randomly sampled and used for each level (depth) of each tree in the ensemble. We used the range (0.01,1)
- Column sample by node: The fraction of features (columns) to be randomly sampled and used for each split (node) of each tree in the ensemble. We used the range (0.01,1)
- Number of estimators: The number of decision trees (estimators) to be trained in the ensemble and helps to balance between model complexity and prediction accuracy by increasing the number of trees until a satisfactory performance is achieved. We used the range (1, 2000).

- lambda: Determines the L2 regularization term (Ridge penalty) applied to the weights of the model. We used the range [0, 100].
- alpha: determines the L1 regularization term (Lasso penalty) applied to the weights of the model. We used the range [0, 100].
- Scale positive weight: Determines the ratio of the number of negative class samples to the number of positive class samples. We used the range [0, 100].
